# Human Cardiac Progenitor Cell-Derived Extracellular Vesicles Exhibit Promising Potential for Supporting Cardiac Repair *in Vitro*

**DOI:** 10.3389/fphys.2022.879046

**Published:** 2022-05-20

**Authors:** Veronica Romano, Immacolata Belviso, Anna Maria Sacco, Domenico Cozzolino, Daria Nurzynska, Cristiano Amarelli, Ciro Maiello, Felice Sirico, Franca Di Meglio, Clotilde Castaldo

**Affiliations:** ^1^ Department of Public Health, University of Naples Federico II, Naples, Italy; ^2^ Department of Medicine, Surgery and Dentistry “Scuola Medica Salernitana”/DIPMED, University of Salerno, Baronissi, Italy; ^3^ Department of Cardiovascular Surgery and Transplant, Monaldi Hospital, Naples, Italy

**Keywords:** extracellular vesicles, human cardiac progenitor cells, human cardiac fibroblasts, cardiac regenerative medicine, paracrine effects

## Abstract

Although human Cardiac Progenitor Cells (hCPCs) are not retained by host myocardium they still improve cardiac function when injected into ischemic heart. Emerging evidence supports the hypothesis that hCPC beneficial effects are induced by paracrine action on resident cells. Extracellular vesicles (EVs) are an intriguing mechanism of cell communication based on the transport and transfer of peptides, lipids, and nucleic acids that have the potential to modulate signaling pathways, cell growth, migration, and proliferation of recipient cells. We hypothesize that EVs are involved in the paracrine effects elicited by hCPCs and held accountable for the response of the infarcted myocardium to hCPC-based cell therapy. To test this theory, we collected EVs released by hCPCs isolated from healthy myocardium and evaluated the effects they elicited when administered to resident hCPC and cardiac fibroblasts (CFs) isolated from patients with post-ischemic end-stage heart failure. Evidence emerging from our study indicated that hCPC-derived EVs impacted upon proliferation and survival of hCPCs residing in the ischemic heart and regulated the synthesis and deposition of extracellular-matrix by CFs. These findings suggest that beneficial effects exerted by hCPC injection are, at least to some extent, ascribable to the delivery of signals conveyed by EVs.

## Introduction

Even if we are far from effective therapies, the positive outcomes of the clinical application of stem cell therapies have been remarkable with thousands of trials currently registered in the National Institutes of Health clinical trials database (www.clinicaltrials.gov). Results from the early phase trials have demonstrated cell therapy as safe, feasible and potentially efficacious in a wide range of diseases and medical fields, from cardiology (Matsa et al., 2014), ischemic stroke (Sinden et al., 2012) and peripheral ischemia (Raval and Losordo, 2013), to cancer (Abd Elmageed et al., 2014; Cieri et al., 2014; Zhou et al., 2017). Despite the enthusiastic results, for many of these therapies, therapeutic benefit cannot be attributed to stem cell survival and differentiation as they are not retained by organs (Gnecchi et al., 2008; Hicks et al., 2013; Katare et al., 2014). The beneficial effects of cell implants without cell survival and retention along with well-researched trophic effects of the cell-conditioned culture medium (Davidson and Yellon, 2018) suggest that secreted paracrine factors may be involved and could be responsible for the observed results (Bi et al., 2007; Parekkadan et al., 2007). The paracrine hypothesis has been strengthened by the discovery that stem cells release not only soluble factors, like cytokines and chemokines, but also extracellular vesicles (EVs) eliciting similar biological activity to the stem cells themselves (Parekkadan et al., 2007; Timmers et al., 2007; Timmers et al., 2011; Camussi et al., 2013; Liang et al., 2014). EVs had gained immense research interest over the last few years due to their promising diagnostic and therapeutic potential (Cossetti et al., 2014) as cell-free approach (Gnecchi et al., 2005; Xia et al., 2018). EVs are vesicles varying from 30 to several thousand nanometers in size and they are considered vesicles of endosomal origin or shed from the plasma membrane secreted by most cell types under normal and pathological conditions (Frühbeis et al., 2012; Barteneva et al., 2013; Regev-Rudzki et al., 2013; Yáñez-Mó et al., 2015). *In vitro* EVs can be isolated from cell-conditioned medium and from different body fluids such as plasma (Caby et al., 2005), urine (Pisitkun et al., 2004) and breast milk (Karlsson et al., 2016).

First simply considered a garbage can, EVs are now accounted as specifically secreted vesicles that enable intercellular communication and have become the focus of exponentially growing interest, both to study their functions and to understand ways to use them in the development of a non-invasive diagnostic and therapeutic tool (Wang et al., 2008; Cantaluppi et al., 2012). In fact, to date EVs are considered a key factor in cell–cell communication (Camussi et al., 2010) and, in addition, they participate in several different functions and in a large variety of pathways, such as immune response (Harrell et al., 2019), cardiovascular protection (Jansen et al., 2017) and cancer (Urabe et al., 2020). It has been demonstrated that mRNA and miRNAs delivered by EVs are translated and regulate gene expression of acceptor cells, influencing their biology (Qiu et al., 2018). They contribute to the maintenance of cardiovascular and arterial homeostasis as well as to pathologies and play a functionally significant role in processes such as immune response, tumor progression, proliferation, and apoptosis (Mittelbrunn and Sanchez-Madrid, 2012; Barile et al., 2014; Xiao et al., 2016; Qiao et al., 2019). Several reports suggested that the myocardial tissue secretes specific microvesicles involved in heterocellular communication in the adult heart (Loyer et al., 2014; Chistiakov et al., 2016) and that common drugs used in cardiac patients may enhance the release of EVs (Casieri et al., 2020). Since EVs are demonstrated to be mediators of extracellular communication, it is extremely likely that they are important communicators of ischemic signaling and myocardial repair too (Barile et al., 2014; Tian et al., 2021). At present, several promising strategies for the application of EVs in cardiovascular therapy are under development.

As data collected over time from researchers worldwide indicate a considerable heterogeneity and dynamism among EVs, particularly referred to their size, cargo and membrane composition, that are strictly dependent from the cell of origin and from the microenvironmental condition, a common agreement among the scientific community is crucial, considering the intense ongoing investigations on EVs.

A major breakthrough in the field has been signed by the founding of the International Society of Extracellular Vesicles (ISEV), which members have been collaborating since 2011 to harmonize the nomenclature and methods to isolate and characterize EVs. Even though numerous features, peculiar for each subgroup of EVs have been proposed, a specific identity fully accepted by the scientific community does not exist yet, and no markers among those found in EVs are considered distinguishing or completely accurate for their characterization. The lack of a standardized method for EVs isolation frequently causes a non-effective or an incomplete purification of the desired particles, producing a complex blend of diverse EVs. Thus, EVs have no consensual nomenclature and they are usually indicated and named based on their cell of origin.

Even though the guidelines established by the ISEV are actually the most accurate and complete and represent a benchmark to give an orientation in the iffy and chaotic universe of EVs, they are hardly applicable for laboratory practice for their complexity, and however, none of the methods mentioned allows the full specificity for the isolation and characterization of EVs (Théry et al., 2018).

**FIGURE 1:**
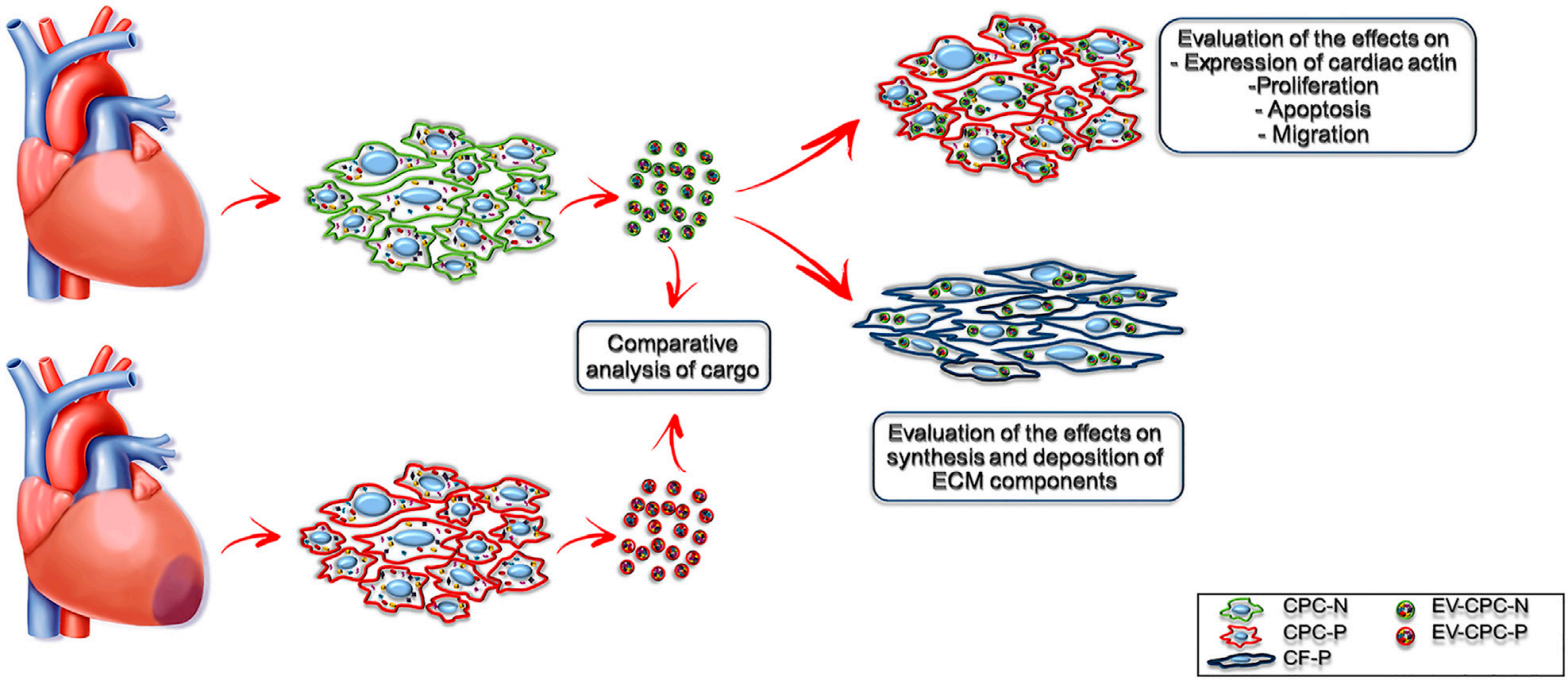
Experimental design. Schematic representation of the study.

Since EVs could be used as nanoparticles for targeted delivery of various bioactive molecules, such as growth factors (Kreimer et al., 2015), mRNA (Ratajczak et al., 2006) miRNAs (Crescitelli et al., 2013) and mitochondria (Gomzikova et al., 2021), we hypothesized that stem/progenitor cell derived EVs have a great therapeutic potential for cardiac repair in regenerative medicine. Aiming at elucidating the underlying mechanism of the beneficial effects of the injection of human Cardiac Progenitor Cells (hCPCs) (Kajstura et al., 2004; Chakravarty et al., 2017; Makkar et al., 2020; Ostovaneh et al., 2021) that are attributable to EVs release, we isolated EVs delivered by CPCs from both normal (EV-CPC-N) and pathological (EV-CPC-P) adult human hearts and, leaving out a comprehensive characterization of EVs and their subpopulations, we restricted the analysis of the signals they carried to growth factors and mRNAs specific for cardiac cell lineages. Further, we investigated their potential to modulate signaling, proliferation, survival, and migration of recipient cells and even to modulate the extracellular compartment, by affecting fibroblast synthesis of extracellular matrix (ECM) proteins (Figure 1).

## Materials and Methods

### Cardiac Tissue Samples

Cardiac tissue samples were obtained from normal and pathological adult human hearts. Samples of atrial appendages from normal hearts (*n* = 11, 6 males and 5 females, mean age 35 ± 12 years) were collected from heart waste fragments of donors who died for reasons other than cardiovascular diseases. Pathological samples were taken from atrial appendages of explanted hearts of patients with end-stage heart failure associated with ischemic cardiomyopathy and undergoing heart transplantation (*n* = 20, 14 males and 6 females, mean age 56 ± 5.5 years, mean ejection fraction 18.75 ± 3.6%) (Supplementary Table S1). All patients provided written informed consent for use of heart tissue for experimental studies and specimens were collected, without patient identifiers, following protocols approved by the Monaldi Hospital and in conformity with the principles outlined in the Declaration of Helsinki. Ethical approval for this study was gained from the University of Naples Federico II ethics committee (reference number 79/17).

### Isolation and Culture of CPCs

Cardiac tissue samples were dissected, minced, and enzymatically disaggregated by incubation in 0.25% trypsin and 0.1% (w/v) collagenase II (both from Sigma-Aldrich, St. Louis, MO, United States), for 30 min at 37°C. The digestion was stopped by adding a double volume of Hank’s balanced salt solution (HBSS) supplemented with 10% fetal bovine serum (FBS) (Sigma-Aldrich). This preparation was further disaggregated by pipetting and tissue debris and cardiomyocytes were removed by sequential centrifugation at 100 g for 2 min, passage through 40 μm cell strainer and centrifugation at 400 g for 5 min. Cell population was seeded on culture dishes in Dulbecco’s Modified Eagle Medium/Nutrient Mixture F-12 (DMEM/F-12) supplemented with 10% FBS, 5% horse serum, 0.2 mM glutathione, 5U/L erythropoietin, 50 μg/ml porcine gelatine, 50 IU/ml Penicillin G-Streptomycin (all from Sigma-Aldrich) and 10 ng/ml basic fibroblast growth factor (bFGF) (Peprotech, Rocky Hill, NJ, United States) and cultured in a humidified incubator at 37°C and 5% CO_2_ in air. The medium was changed every 3 days to remove cell debris and maintain a physiological pH. Once the adherent cells were more than 75% confluent, they were detached with 0.25% trypsin-EDTA (Sigma-Aldrich) and cell suspension was used to isolate CPCs by immunomagnetic cell sorting (Di Meglio et al., 2007; Castaldo et al., 2008; Di Meglio et al., 2010a; Di Meglio et al., 2010b; Castaldo et al., 2013; Nurzynska et al., 2013).

Both CPC from normal (CPC-N) and pathological hearts (CPC-P) were pooled to reduce the biological variability among patients (Metzger et al., 2020) and plated at a 2 × 10^4^ /cm^2^ density and cultured with Nutrient Mixture F-12 medium, supplemented with 10% FBS, 5% horse serum, 0.2 mM glutathione, 5 U/L erythropoietin, 50 μg/ml porcine gelatin, 50 IU/ml penicillin G-streptomycin (all from Sigma-Aldrich) and 10 ng/ml bFGF (Peprotech). Culture dishes were checked daily at a phase contrast microscope (Olympus, Tokyo, Japan) and the medium was replaced every 3 days to remove cell debris and maintain a physiological pH.

### Isolation and Culture of Cardiac Fibroblasts

Fibroblasts from the hearts of patients with end-stage heart failure associated with ischemic cardiomyopathy (CF-P) were isolated by outgrowth as previously described (Pagano et al., 2017; Sacco et al., 2019; Belviso et al., 2020a). Briefly, small fragments of myocardium were placed in culture plates under sterile coverglasses and incubated with Dulbecco’s Modified Eagle Medium (DMEM) supplemented with 10% FBS and 0.5% penicillin and streptomycin (all from Sigma-Aldrich) in a humidified incubator at 37°C and 5% CO_2_ in air. After a time ranging between 7- and 21-days cells outgrew from fragments. Coverglasses were removed and fibroblasts were pooled and passaged. Culture dishes were checked daily at a phase contrast microscope (Olympus) and the medium was replaced every 3 days to remove cell debris and maintain a physiological pH.

### EVs Isolation

Once CPC-N and CPC-P were 80–90% confluent, complete medium was replaced with a serum-free medium, which was collected after 48 h and processed to isolate EVs. The isolation was carried out by ExoQuick-TCTM (System Biosciences, Mountain View, CA, United States), following the protocol supplied with the reagent without introducing any modification. Briefly, culture medium from each cell population was pooled and centrifuged at 3000 g for 15 min at 4°C to eliminate cells and cellular debris. The supernatant was transferred to sterile tubes adding 1 ml of ExoQuick-TCTM Exosome Isolation Reagent to each 5 ml of cell culture medium. Tubes were mildly agitated until the separation between the two phases was no longer visible, then incubated at 4°C overnight. The next day tubes with ExoQuick-TC/culture medium mixture were centrifuged at 1500 g for 30 min at 4°C to allow the precipitation of the white/beige pellet. Then the supernatant was discarded, and the residual ExoQuick-TC solution was spun down by centrifugation at 1500 g for 5 min at 4°C. The EVs were quantified using Bradford Assay (Gupta et al., 2021), then pooled and stored at −80°C until their use for specific assays (Belviso et al., 2016).

### Growth Factor Array

Pellet obtained from both EV-CPC-P and EV-CPC-N was lysed using RIPA buffer containing Tris-HCl 50 mM pH 7.4, NaCl 150 mM, Nonidet P-40 1%, sodium deoxycholate 0.25%, Na_3_VO_4_ 1 mM and NaF 1 mM (all from Sigma-Aldrich) supplemented with protease inhibitor cocktails complete ULTRA Tablets, Mini, EASYpack (Roche Diagnostics Corporation, Mannheim, Germany). Protein concentration was determined by Bio-Rad Protein Assay (Bio-Rad Laboratories, Hercules, CA, United States) using albumin from bovine serum (Sigma-Aldrich) as a standard. Protein array to simultaneously detect 41 targets between growth factors and growth factor receptors was performed on both EV-CPC-P and EV-CPC-N lysates, using Human Growth Factor Antibody Array C1 kit (Raybiotech, Norcross, GA, United States). Briefly, the array membranes binding primary antibodies were blocked for 30 min and then incubated for 2.5 h with 100 µg of protein for each sample. Both steps were performed at room temperature. After washing, membranes were incubated with a cocktail of biotin-conjugated antibodies overnight at 4°C, and then with horseradish peroxidase (HRP)-conjugated streptavidin for 2 h at room temperature. Membranes were then developed, and signal was detected by chemiluminescence and autoradiography. Numerical comparison of the signal densities of growth factors was performed strictly according to the guidelines for data extraction supplied with the array protocol. Briefly, spot signal densities from the scanned images of arrays were obtained using ImageJ densitometry software (https://imagej.nih.gov/ij/download.html). The background was then subtracted from the densitometry data, and the obtained values were normalized to the positive control signals. Data were expressed as the mean ± SEM (Supplementary Table S2).

### RNA Extraction and Quantitative Real-Time PCR From EV-CPC-N and EV-CPC-P

Total RNA was extracted from EVs isolated from both CPC-N (*n* = 11) and CPC-P (*n* = 20), combining Trizol Reagent (Invitrogen, Thermo Fisher Scientific, Carlsbad, CA, United States) and RNeasy Micro kit (Qiagen, Hilden, Germany), as previously described (Di Meglio et al., 2010c). RNA obtained was resuspended in RNase-free water and the final concentration was determined by spectrophotometric analysis with Nanodrop 2000 (Thermo Scientific). RNA extracted was then retrotranscribed with QuantiTect Reverse Transcription kit (Qiagen) following the protocol provided by the supplier as previously described (Putame et al., 2020). In order to assess the presence and the quantity of specific transcripts, a Real-Time PCR was performed using PrecisionPLUS^TM^ MasterMix kit (Primerdesign, Southampton, United Kingdom) according to manufacturer’s protocol. The detection was carried out by measuring the binding of the fluorescent dye SYBR Green I to double-stranded DNA. DNA was then amplified using Mastercycler ep realplex^4S^ (Eppendorf, Hamburg, Germany). All samples were tested in triplicate and comparative quantification of target genes expression was based on cycle threshold (Ct) (Livak and Schmittgen, 2001). All the primers used, listed in Table 1, were designed with Primer3 software (http://frodo.wi.mit.edu) starting from the coding sequence of mature mRNA available on GeneBank. Melt curve analysis was used to assess amplification of non-specific products, uniformity of product and formation of primer dimers. Data were expressed as the mean ± SEM.

**TABLE 1:** List of primer sequences used for Real-time PCR on EV-CPC-N and EV-CPC-P.

Gene	Forward primer	Amplicon length (nt)
	Reverse primer	
GAPDH	5′-CTC​TCT​GCT​CCT​CCT​GTT​CG-3′	114
	5′-ACG​ACC​AAA​TCC​GTT​GAC​TC-3′	
ACTA2	5′-CTG​AGC​GTG​GCT​ATT​CCT​TC-3′	133
	5′-CTG​AGC​GTG​GCT​ATT​CCT​TC-3	
ACTC1	5′-TCG​GGA​CCT​CAC​TGA​CTA​CC-3′	125
	5′-CAA​AAT​CCA​GGG​CGA​CAT​AG-3′	
CD106 or VCAM1	5′-AAA​ATG​GAA​GAT​TCT​GGG​GTT-3′	134
	5′-TTG​ACA​CTC​TCA​GAA​GGA​AAA​GC-3′	
CD90 or THY1	5′-CTA​GTG​GAC​CAG​AGC​CTT​CG-3′	198
	5′-GCC​CTC​ACA​CTT​GAC​CAG​TT-3′	
ETS1	5′-TGG​GGA​CAT​CTT​ATG​GGA​AC-3′	88
	5′-TGG​ATA​GGC​TGG​GTT​GAC​TC-3′	
FVIII	5′-GCT​CTG​GGA​TTA​TGG​GAT​GA-3′	80
	5′-TCT​TGA​ACT​GAG​GGA​CAC​TGC-3′	
GATA6	5′-GTG​TGC​AAT​GCT​TGT​GGA​CT-3′	103
	5′-TGT​TCT​TAG​GTT​TTC​GTT​TCC​TG-3′	
MEF2C	5′-AGG​CAG​CAA​GAA​TAC​GAT​GC-3′	88
	5′-TAC​GGA​AAC​CAC​TGG​GGT​AG-3′	
NKX2.5	5′-ACT​TGA​ATG​CGG​TTC​AGA​GC-3′	137
	5′-GAG​TCA​GGG​AGC​TGT​TGA​GG-3′	
SOX9	5′-CCA​ACG​CCA​TCT​TCA​AGG-3′	141
	5′-GCT​GCA​CGT​CGG​TTT​TG-3′	

### *In vitro* Administration of EVs

EV-CPC-N were used to prepare a conditioned medium (EV-CM), to test effects produced by EVs on both CPC-P and CF-P. EV-CPC-N were resuspended in the medium specific for each cell population and added to CPC-P and CF-P culture dishes at the final concentration of 0.1 mg/ml (Barile et al., 2014) and cultured for 48 h. During the culture, the morphology of cells was evaluated by phase contrast microscope observation with Nikon Eclipse T*i*-E DS-Qi2 Microscope (Nikon Corporation, Tokyo, Japan).

### RNA Extraction and Quantitative Real-Time PCR From CPC-P and CF-P

Total RNA extracted from CPC-P and CF-P treated or not with EV-CPC-N was retrotranscribed and Real-Time PCR was performed as previously described. All the primers used are listed in Table 2.

**TABLE 2:** List of primer sequences used for Real-time PCR on CPC-P and CF-P.

Gene	Forward primer	Amplicon length (nt)
	Reverse primer	
GAPDH	5′-CTC​TCT​GCT​CCT​CCT​GTT​CG-3′	114
	5′-ACG​ACC​AAA​TCC​GTT​GAC​TC-3′	
ACTC1	5′-TCG​GGA​CCT​CAC​TGA​CTA​CC-3′	125
	5′-CAA​AAT​CCA​GGG​CGA​CAT​AG-3′	
COL1A1	5′-GTG​AAC​AAG​GTC​CCT​CTG​GA-3′	92
	5′-ACC​GTT​GAG​TCC​ATC​TTT​GC-3′	
COL3A1	5′-TGT​GAA​TCA​TGC​CCT​ACT​GG-3′	80
	5′-CCT​ACT​GCT​ACT​CCA​GAC​TTG​ACA-3′	
COL4A1	5′-CTC​TGG​CTG​TGG​CAA​ATG​T-3′	92
	5′-CAG​GAA​ACC​CAA​TGA​CAC​CT-3′	
FN1	5′-ACC​GAG​GTG​ACT​GAG​ACC​AC-3′	137
	5′-GAC​ACA​ACG​ATG​CTT​CCT​GA-3′	
LAMA2	5′-GGG​TTA​GGG​AGC​AAA​AAT​G-3′	132
	5′-TCG​CAG​CCT​TTC​CAA​TTA​TC-3′	
TNC	5′-GCT​CAA​AGC​AGC​CAC​TCA​TT-3′	108
	5′-CCC​ATA​TCT​GGA​ACC​TCC​TCT-3′	

### Immunofluorescence

To evaluate the effects of EV-CPC-N, CPC-P and CF-P cultured with EV-CM or with regular medium (control groups) were fixed in 4% paraformaldehyde for 20 min at room temperature after 3 days of culture. After blocking with 10% donkey serum, cells were incubated with primary antibody against human α-sarcomeric actin (Di Meglio et al., 2010b) or fibronectin (both from Sigma-Aldrich), for 1 h at 37°C, then washed and incubated with secondary antibody conjugated with fluorescein (Jackson ImmunoResearch Europe, Newmarket, United Kingdom) at 37°C, for 1 h. Nuclei were counterstained with 4′-6-diamidino-2-phenylindole (DAPI) (Merck Millipore) and stained area of culture dishes was mounted in Vectashield (Vector Labs). Microscopic analysis was performed with Nikon Eclipse Ti-E DS-Qi2 Microscope (Nikon) and marker expression was quantified by three independent observers by Nikon Imaging Analytical Software (NIS Elements Analysis 4.50) (Nikon) and expressed as mean fluorescence intensity ± SEM.

### Proliferation and Apoptosis of CPCs

To evaluate proliferation and apoptosis, both CPC-P receiving EV-CM and control group were plated at a density of 2.5 × 10^4^ /cm^2^ and stained with Cell-Clock Cell Cycle Assay and Cell-ApoPercentage Apoptosis Assay (both from Biocolor Ltd., Carrickfergus, United Kingdom), respectively, strictly following manufacturer’s instructions. In the first assay, cells were incubated at 37°C for 1 h with the redox dye supplied by the kit. Such dye is taken up by live cells and its uptake induces a distinct change in cell color, from light green to blue, specifically associated with G1- S- G2- or M-phase of the cell cycle. For ApoPercentage Apoptosis Assay, CPC-P receiving EV-CM or from control group, were first incubated with 3% hydrogen peroxide in the complete medium for 12 h, then stained with the ApoPercentage dye that is selectively imported by cells undergoing apoptosis. Microscopic analysis and quantification were performed by three independent observers with a Leica DM2000 LED microscope (Leica Microsystems) equipped with a digital camera Leica ICC50 HD (Leica Microsystems). Data were expressed as mean percentage of cycling cells over total cells ± SEM, for Cell-Clock Cell Cycle Assay, and as mean percentage of stained cells ± SEM over total cells for Cell-ApoPercentage Apoptosis Assay.

### Migration of CPCs

To evaluate the speed of migration of CPC-P cultured with EV-CM and of CPC-P of control group, scratch wound assay was performed as previously described (Castaldo et al., 2013). Briefly, cells were grown to confluence and a thin scratch was produced in straight line on culture plates with a 10 µl sterile pipette tip, leaving a cell-free zone. Cell culture dishes were first washed with medium to remove debris and then fresh medium was pipetted in the dishes. Plates were placed under Nikon Eclipse T*i*-E DS-Qi2 Microscope (Nikon) equipped with stage incubator (Okolab, Pozzuoli, Italy) and the migration was documented acquiring pictures every 10 min for 8 h by digital camera (Nikon). Data were analyzed by NIS Elements software (Nikon) and expressed as mean speed of migration ± SEM.

### Statistical Analysis

All experiments were performed at least in triplicate, each sample was tested three to five times, and all numerical data were expressed as mean ± SEM. Statistical differences between groups were evaluated with Student’s two-tailed unpaired t-test. A value of *p* ≤ 0.05 was considered statistically significant.

## Results

### Signals Carried by EV-CPC

**FIGURE 2:**
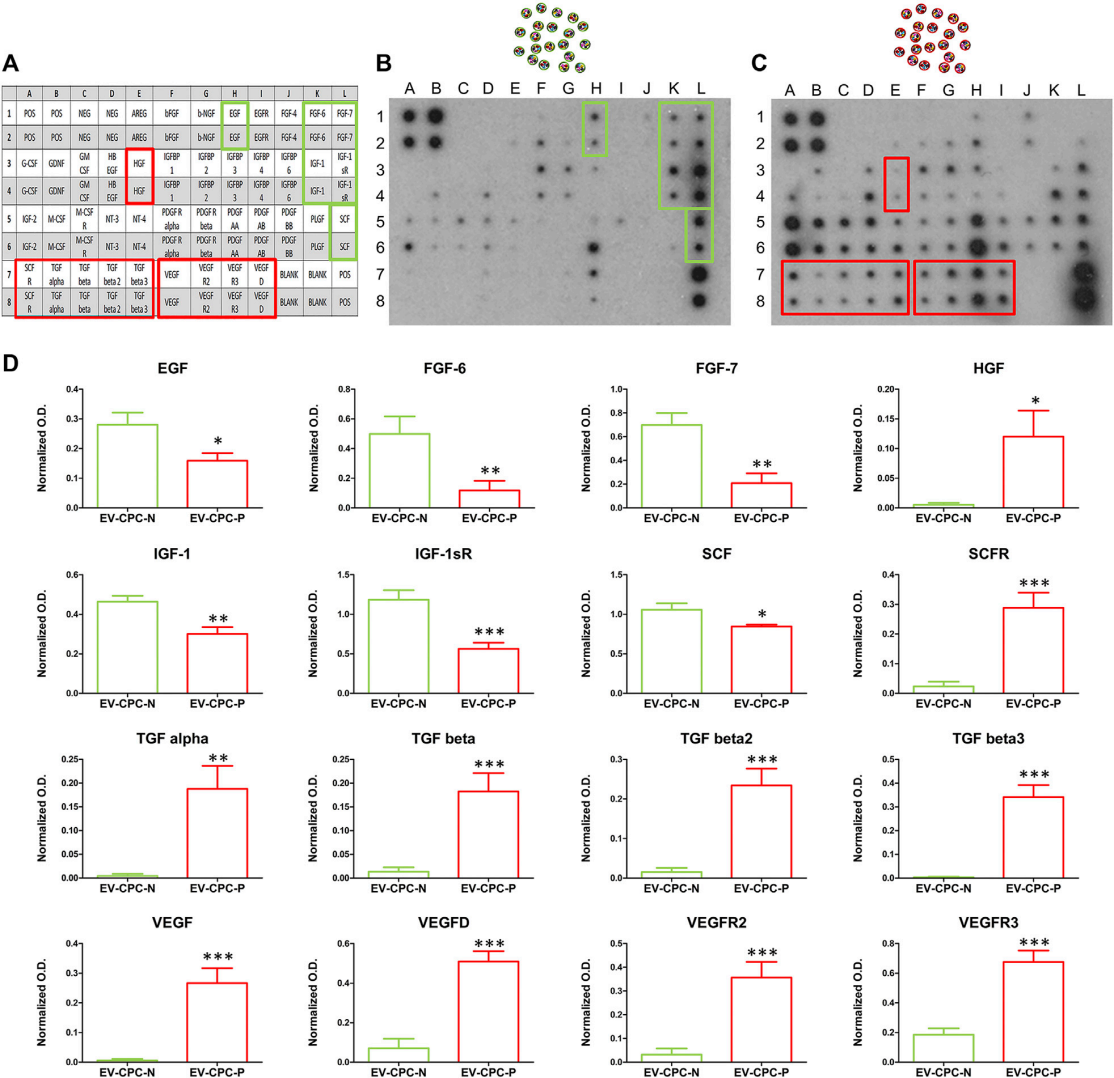
Comparative analysis and quantification of growth factor content in EV-CPC-N and EV-CPC-P cargos. Array map is used as a reference **(A)** and on protein array membranes are highlighted higher amounts of EGF, FGF, IGF-1, and SCF **(D)** in EV-CPC-N **(B)** or higher amounts of HGF, SCF-R, TGF, VEGF, and its receptors **(D)** in EV-CPC-P **(C)**.

Protein array performed to analyze growth factor cargo of EV-CPC revealed differences in the content of growth factors (GFs) between EV-CPC-N and EV-CPC-P. The comparison clearly showed that EV-CPC-N carried factors mostly involved in biological processes like cell migration, proliferation, and differentiation as EGF (Epidermal Growth Factor), FGF-6 and FGF-7 (Fibroblasts Growth Factors-6 and 7). Further, they showed a considerably higher amount of IGF-1 (Insulin-like Growth Factor) and SCF (Stem Cell Factor). On the contrary, EV-CPC-N did not carry factors involved in myofibroblast activation and angiogenesis processes, as TGF-β (Transforming Growth Factor), HGF (Hepatocyte Growth Factor) and VEGF (Vascular Endothelial Growth Factor) that were clearly present in EV-CPC-P. Additionally, a higher expression of receptors VEGFR2, VEGFR3, and SCFR in EV-CPC-P emerged. The assay did not reveal, in EV-CPC-N, the presence of receptors like SCFR, EGFR, PDGFRα, and PDGFRβ (Figure 2). Using the array map as a reference (Figure 2A), the representative images of the protein array membranes clearly showed that EV-CPC-N (Figure 2B) carried higher amounts of EGF, FGF, IGF-1, and SCF (Figure 2D), while EV-CPC-P (Figure 2C) were enriched with HGF, SCF-R, TGF, VEGF and its receptors (Figure 2D).

**FIGURE 3:**
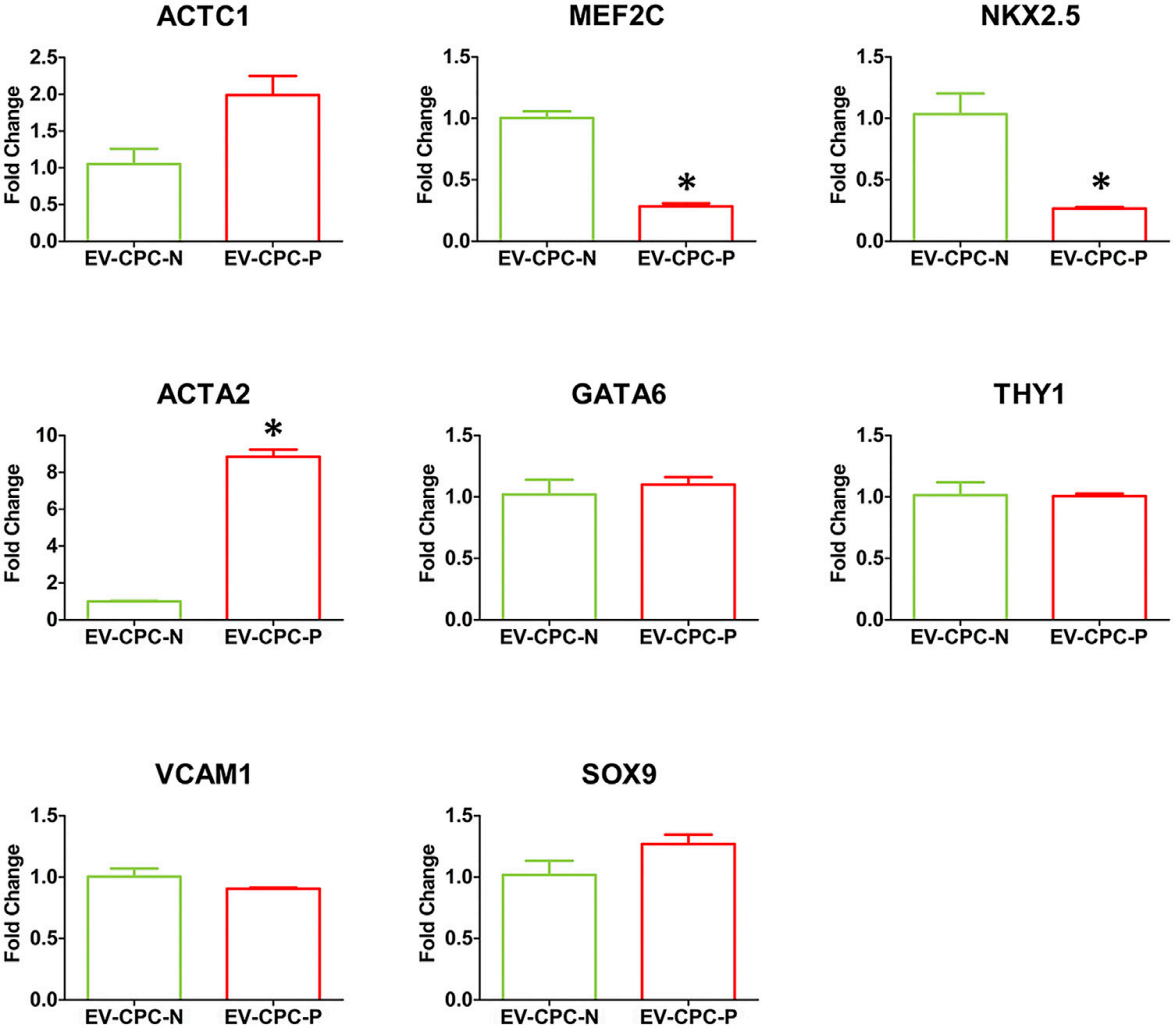
Gene expression analysis of cardiac cell markers in EV-CPC-N and EV-CPC-P cargos. Real-time PCR analysis of the gene expression for markers characteristic of cardiac myocytes, smooth muscle cells and mesenchymal cells showed a downregulated transcription of the cardiac myocyte markers MEF2C and NKX2.5, and an upregulated transcription of the smooth muscle cell marker ACTA2 in EV-CPC-P. (*p ≤ 0.05 vs. EV-CPC-N).

Real-time PCR analysis revealed also that EV-CPC-N and EV-CPC-P transported specific transcripts for cardiac cell progenitors or precursors of cardiomyocyte, as ACTC1, MEF2C, and NKX2.5, and smooth muscle cells (SMCs), as ACTA2 and GATA6. They also carried THY1, VCAM1 and SOX9 (Figure 3), markers of mesenchymal cells. Gene expression analysis revealed differences between EV-CPC-P and EV-CPC-N cargo. Precisely, statistically significant differences (*p* < 0.05) emerged for MEF2C and NKX2.5 transcripts, mainly carried by EV-CPC-N, and for ACTA2 contained in a higher amount in EV-CPC-P (Figure 3).

### Effects of EV-CM on CPC-P

**FIGURE 4:**
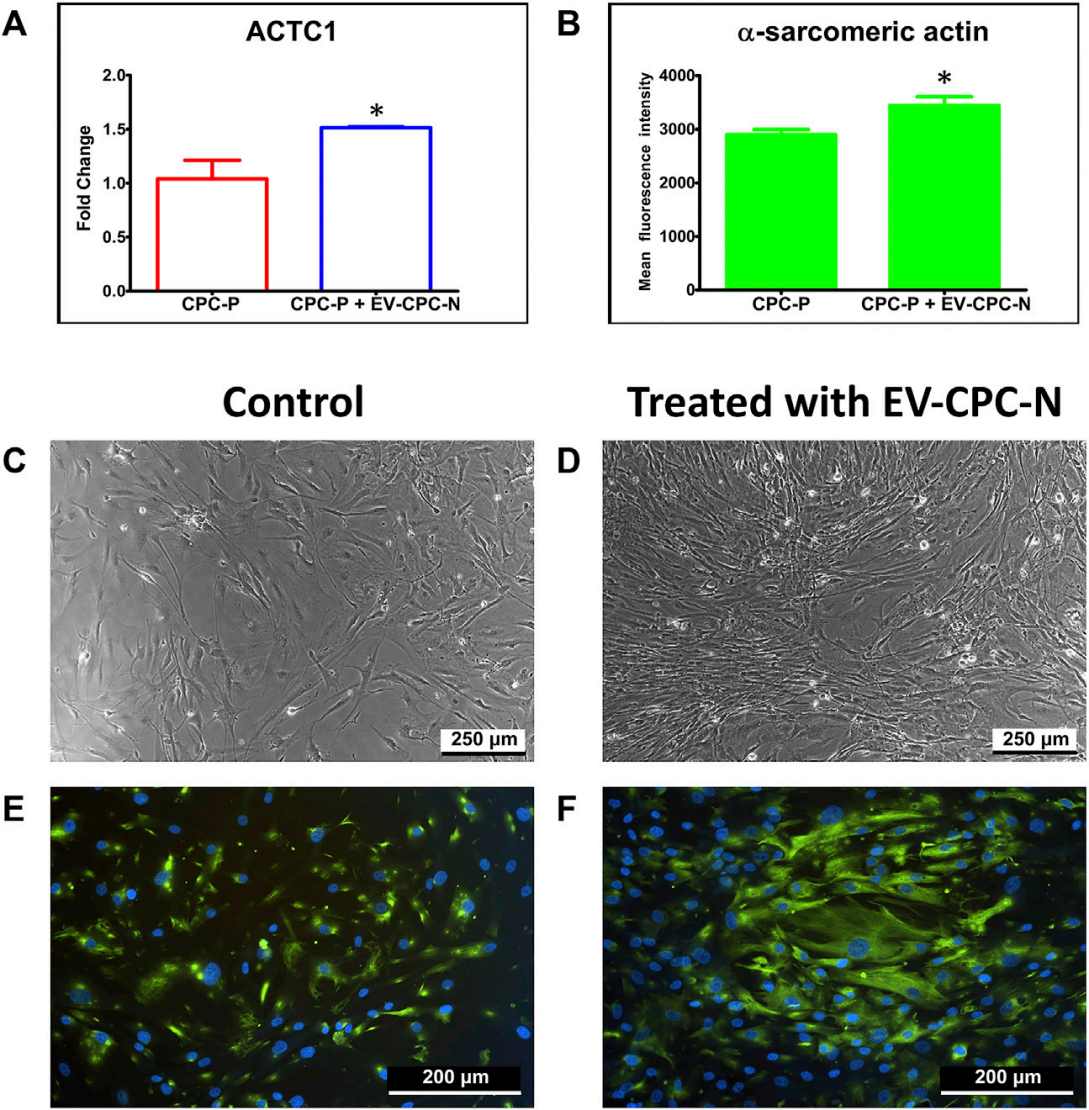
Analysis of α-sarcomeric actin expression at gene or protein level of CPC-P cultured in the absence or in the presence of EV-CPC-N. Graphical representation of ACTC1 expression by Real-time PCR **(A)** and quantification of the α-sarcomeric actin immunopositivity **(B)** in CPC-P cultured with EV-CPC-N vs. CPC-P cultured without EV-CPC-N (*p ≤ 0.05 vs. CPC-P). Analysis of the morphology and α-sarcomeric actin expression of CPC-P cultured in the absence or in the presence of EV-CPC-N. **(C)** and **(D)** Representative images acquired at the phase contrast microscope showing a similar morphology between CPC-P cultured without **(C)** or with **(D)** EV-CPC-N. E and **(F)** Representative images of immunofluorescence analysis showing by the green fluorescence the α-sarcomeric actin expression and distribution pattern in CPC-P cultured without **(E)** or with **(F)** EV-CPC-N. The blue fluorescence is the result of nuclear counterstaining with DAPI. Scale bar: 250 μm for **(C)** and **(D)**, and 200 μm for **(E)** and **(F)**.

Real-time PCR analysis showed an up-regulation of ACTC1 gene, encoding for α-sarcomeric actin, in CPC-P treated with EV-CM with the respect to the control group (Figure 4A).

Observation by phase contrast microscope showed a similar morphology for CPC-P cultured with a regular medium (control group) or with EV-CM (Figures 4C,D). Immunofluorescence supported the Real-time PCR results, revealing that both populations expressed α-sarcomeric actin, even though CPC-P treated with EV-CM showed a significantly (*p* < 0.05) stronger immunopositivity (3442.15 ± 164.2 mean fluorescence intensity in CPC in EV-CM versus 2899.41 ± 96.73 mean fluorescence intensity in control CPC-P) (Figure 4B) and a better organized α-actin pattern and distribution (Figures 4E,F).

**FIGURE 5:**
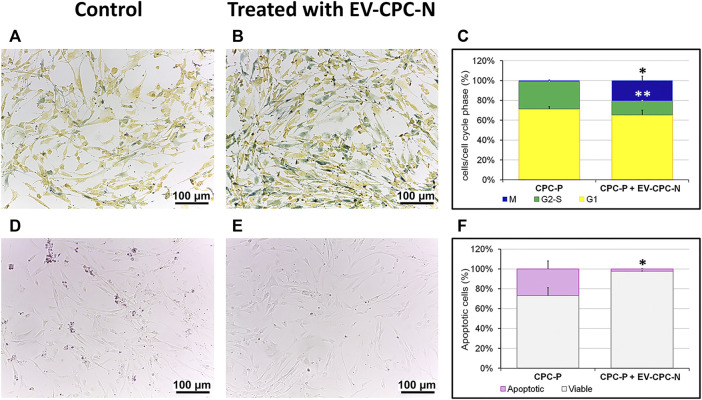
Evaluation of the proliferation and apoptotic rate of CPC-P cultured in the absence or in the presence of EV-CPC-N. **(A–C)**: Representative light microscopy images of the live CPC-P **(A)** and CPC-P cultured with EV-CPC-N **(B)** detection in the G1 (yellow), G2-S (green) or M (blue) phases of the cell cycle and their quantification **(C)** using the Cell-Clock Cell Cycle Assay. **(D–F)**: Representative light microscopy images of the detection of CPC-P apoptosis (purple-red colored cells) as related to the absence **(D)** or the presence **(E)** of EV-CPC-N and quantification of apoptotic rate **(F)** using the Cell-APOPercentage Apoptosis Assay. Asterisks are indicators of the *p* value as follows: significant (*p ≤ 0.05 vs. CPC-P) and very significant (**p ≤ 0.01 vs. CPC-P). Scale bar: 100 μm.

The presence of EV-CPC-N had also effects on proliferation and apoptosis of cells. CPC-P treated with EV-CM showed higher proliferation rate when compared to control group, as indicated by the ratio of cells in S to M phases (Figures 5A,B). Indeed, in the presence of EV-CM the proportion of proliferating cells in M phase reached a percentage of 20.52 ± 4.43%, while the control group showed a significantly lower number of proliferating cells in the same phase, equal to 1.068 ± 0.79%. The percentage of cells in G2-S phase was 14.19 ± 0.63% in CPC-P treated with EV-CM, against a 27.48 ± 2.13% observed in CPC-P in culture with regular medium. No statistically significant differences were observed for cells in G1 phase that showed a percentage of 65.28 ± 5.04 and 71.45 ± 2.42% for CPC-P treated with EV-CM and control group, respectively (Figure 5C). From microscopic analysis a protective effect on oxidative stress-induced apoptosis exerted by EV-CM culture of CPCs also emerged, as demonstrated by the significantly lower percentage of nuclei stained with the dye selective for apoptotic cells (Figures 5D,E). In fact, oxidative stress induced apoptosis in only 2.25 ± 0.31% in case of CPC-P that received EV-CM *in vitro*, while CPC-P cultured in regular medium resulted more susceptible to apoptosis showing a dramatically higher percentage of apoptotic nuclei (26.74 ± 8.08%) (Figure 5F).

**FIGURE 6:**
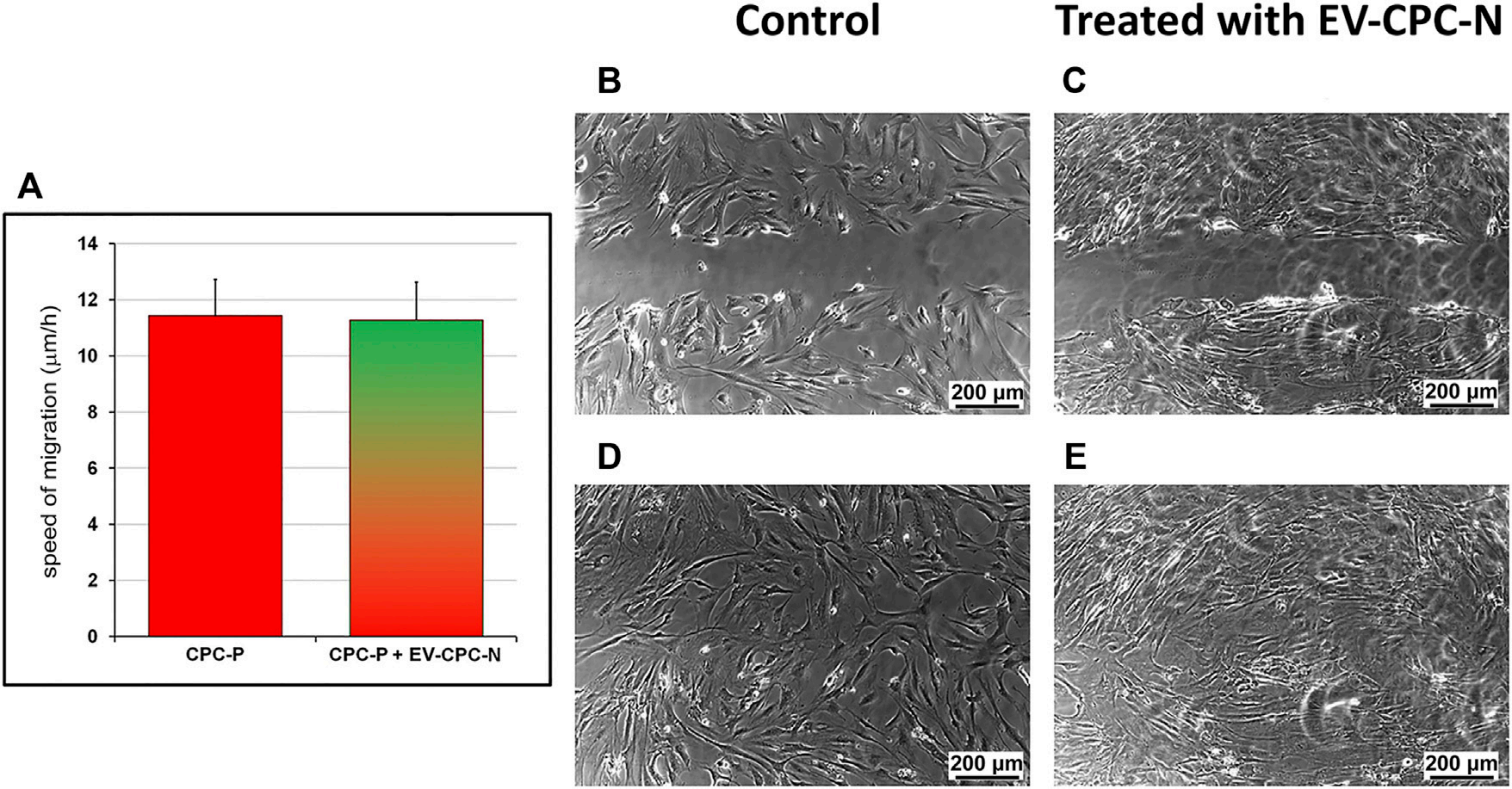
Evaluation of the speed of migration of CPC-P cultured in the absence or in the presence of EV-CPC-N. The scratch wound assay allowed the measurement of cell migration speed. Migration was not affected by administration of EV-CPC-N **(A)** and complete closure of scratch wound made in CPC-P adherent monolayer cultured in the absence **(B)** or in the presence **(C)** of EV-CPC-N occurred within 8 h [**(D)** and **(E)**, respectively]. Scale bar: 200 μm.

After the scratch wound assay, cell migration was monitored in real time by time-lapse imaging which also yielded valuable cell morphology and localization. Images from scratched areas of cell monolayers were recorded every 10 min for 8 h. Results obtained indicated that migration was not affected by administration of EV-CM, as the speed of migration did not differ significantly between CPC-P treated with EVs (11.28 ± 1.34 μm/h) and control group (11.43 ± 1.31 μm/h), and complete wound healing occurred after 8 h in both CPC populations (Figure 6).

### Effects of EV-CM on CF-P

**FIGURE 7:**
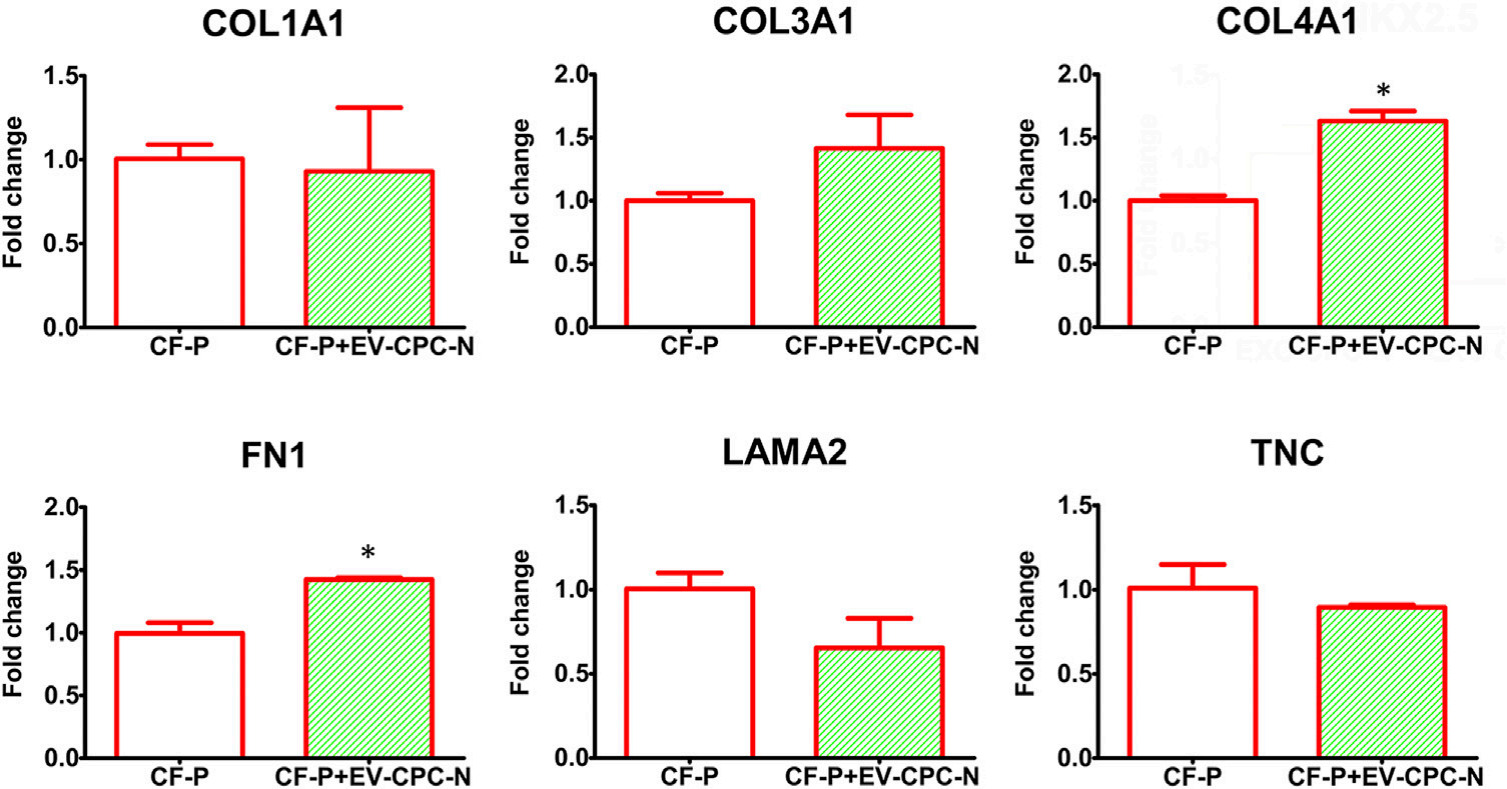
Gene expression analysis of specific transcription of ECM proteins in CF-P cultured in absence or in the presence of EV-CPC-N. Real-time PCR analysis showed a significant up-regulation of genes encoding for collagen type IV and fibronectin. No statistically significant differences emerged for laminin, tenascin, collagen type I and type III (*p ≤ 0.05 vs. CF-P).

**FIGURE 8:**
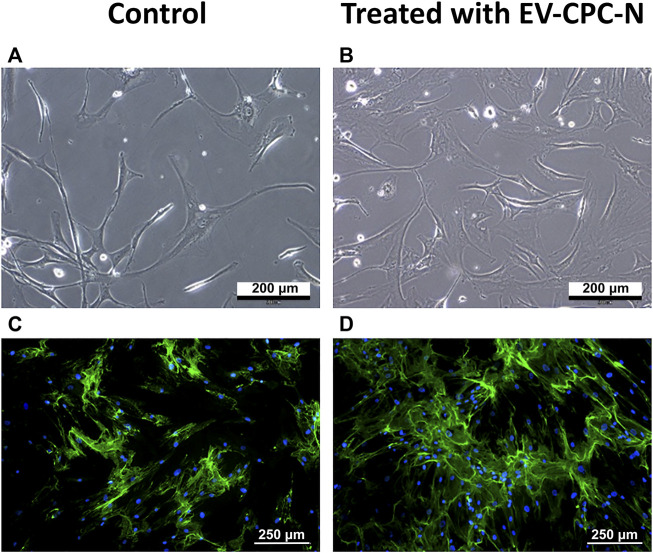
Microscopic analysis of the morphology and immunopositivity for fibronectin of CF-P cultured in the absence or in the presence of EV-CPC-N. **(A)** and **(B)** representative images acquired at phase contrast microscope showing the morphology of CF-P cultured in the absence **(A)** or in the presence **(B)** of EV-CPC-N. **(C)** and **(D)** Representative images of immunofluorescence analysis showing by the green fluorescence the fibronectin distribution pattern in CF-P cultured in the absence **(C)** or in the presence **(D)** of EV-CPC-N. The blue fluorescence is the result of nuclear counterstaining with DAPI. Scale bar: 200 μm for A and B, 250 μm for **(C)** and **(D)**.

Real-time PCR analysis revealed that CF-P cultured with EV-CM showed a significant up-regulation of genes encoding for specific ECM proteins, like collagen type IV and fibronectin, while no statistically significant differences emerged for laminin, tenascin, collagen type I and type III with respect to the control group (Figure 7). At the microscopic observation, CF-P cultured with EV-CM exhibited less elongated morphology with respect to the control group (Figures 8A,B). Fibronectin expression was clearly extracellular, and its synthesis and deposition increased after EV-CM administration to CF-P (1118.96 ± 118.5 versus 2349.31 ± 261.0 mean fluorescence intensity in control CF-P and in CF-P cultured with EV-CM, respectively; *p* = 0.005). Furthermore, fibronectin was secreted and assembled into distinctive fibrillary form that resulted homogeneously distributed in CF-P cultured in EV-CM with respect to the control group where the protein was fragmented and disarranged (Figures 8C,D).

## Discussion

The hypothesis that stem cells could exert therapeutic activity through their secretions is highly plausible as stem cell secretions are known to include many biologically potent molecules such as growth factors, cytokines, chemokines, and bioactive lipids that could elicit wide-ranging physiological effects (Postiglione et al., 2003; Anthony and Shiels, 2013). Growing evidence suggests that adult stem/progenitor cells may exert dramatic effects on the repair of various tissues through secreted factors including EVs (Maguire, 2013; Castaldo and Chimenti, 2018). Indeed, up to 80% of the therapeutic activities of adult stem/progenitor cells injected in ischemic hearts have been shown to occur through paracrine mediated effects (Chimenti et al., 2010), giving rise to the novel notion of stem cell therapy without cells (Maguire, 2013). EVs are secreted by cardiac and vascular cells and stem cells in culture (Barile et al., 2014; Akyurekli et al., 2015; Lamichhane et al., 2014).

To the best of our knowledge, this is the first study comparing the EVs cargo released by CPC isolated directly from normal and pathological adult human hearts of patients with end-stage heart failure due to chronic ischemic heart disease and investigating their effects on both cellular and extracellular compartment *in vitro* (Figure 1). Evidence emerging from the present study highlights a remarkable difference between the content of EV-CPC-N and EV-CPC-P (Figures 2, 3), suggesting that EVs secreted by CPC-N could contribute to the beneficial effects elicited by CPC injection through the delivery of their cargo and hold great promise to become a therapeutic option to treat cardiac diseases. As a matter of fact, we found that EV-CPC-N carry growth factors like EGF, FGF, IGF-1 that are involved in biological processes like cell proliferation, development, differentiation, and migration (Schuldiner et al., 2000), and SCF, exerting a crucial role in maintenance of stem cell compartment. All these factors could potentially boost cardiac repair mainly through endogenous stem cell homing and activation. EV-CPC-P, instead, transport considerably higher amount of growth factors mainly involved in the induction of fibroblast transdifferentiation towards myofibroblasts (TGF-β and HGF), strengthening the hypothesis that cardiac disease affects CPC compartment too (Nurzynska et al., 2013), and that the release of pro-fibrotic factors by EV-CPC-P could be involved in sustaining the pathological remodeling affecting the ischemic heart. However, CPC-P transport higher amounts of SCFR that ensures promptness to respond to SCF stimulation and to EV-CPC-N cargo enriched with SCF. Notably, considering the ECM functions as storage for growth factors (Di Meglio et al., 2017; Belviso et al., 2020b), the delivery of EV-CPC-N in post-ischemic myocardium would imply the delivery and storage of SCF in cardiac ECM, resulting, in turn, in the expansion of CPC resident population expressing SCF-R. Moreover, EV-CPC-P cargo is enriched with VEGF and its receptors (VEGFR2 and VEGFR3) that are involved in the stimulation of angiogenesis, essential to prevent heart failure through the control of cardiomyocyte hypertrophy and contractility (Gogiraju et al., 2019; Di Meglio et al., 2012) and to improve cardiac function (Dougherty et al., 2020). In addition, EVs are lipid vesicles, which represent ideal vehicles to deliver also genetic materials, such as mRNA, from one cell to another. In recent years, many published reports suggest that in addition to cancer (Zhou et al., 2017; Abd Elmageed et al., 2014) and neurodegenerative disorders (Izadpanah et al., 2018), major cardiovascular and metabolic pathologies like coronary artery disease, myocardial infarction and heart failure are highly influenced by the EV directed transfer of molecules (Femminò et al., 2020; Cheng et al., 2014). It has been demonstrated that mRNAs and miRNAs delivered by EVs are translated and regulate gene expression of acceptor cells influencing their biology (Mitchelson and Qin., 2015; Corsten et al., 2010). A primary obstacle to functional recovery of the infarcted or failing human heart is the limited proliferative capacity of cardiomyocytes and their insufficient mechanism for regeneration. The loss of cardiomyocytes after injury, in fact, cannot be compensated and a strategy to replace them could be inducing resident CPC to differentiate into new, healthy cardiomyocytes (Di Meglio et al., 2010a). Our findings by Real-time PCR analysis of EV-CPC cargo revealed the presence of transcripts specific for cardiac or mesenchymal differentiation in EV-CPC-N as well as in EV-CPC-P and an up-regulation of the transcription of cardiac myocyte specific transcription factors in EV-CPC-N, like NKX2.5 and MEF2C. Accordingly, when delivered to CPC-P, EV-CPC-N induced an increased expression of α-sarcomeric actin (Figure 4). Instead, consistently with the evidence of a cargo enriched with pro-angiogenic factors, by Real-time PCR analysis of EV-CPC-P we found signals inducing mostly vascular smooth muscle cell differentiation, as they expressed high levels of ACTA2 (Figure 3). The effect observed in controlling gene expression levels in CPC-P following their interaction with EV-CPC-N is still to get acquainted in detail. Based on our knowledge the cellular response to EV-CPC-N administration could be elicited both by a stimulation of gene transcription in CPC-P, or even by a direct transfer of mRNAs transported by EVs. EVs from CPC have also been reported to inhibit cardiomyocyte apoptosis and to improve cardiac function after myocardial infarction (MI) in animal models, ameliorating the deleterious consequences of myocardial ischemia, and enhancing cardiogenesis (Barile et al., 2014; Khan et al., 2015; Ciullo et al., 2019). Here we tested the effects exerted by EV-CPC-N on capacity of migration, proliferation, and susceptibility to apoptosis of CPC-P, to elucidate the underlying mechanisms responsible for positive outcomes of CPC-based therapy and in the view of a possible cell-free therapeutic approach employing EV-CPC-N to stimulate and support the resident CPC population. Although a similar speed of migration was observed for CPC-P either in the presence or absence of EV-CPC-N (Figure 6), probably due to growth factors enhancing migration like IGF1 and FGF6 that were found in EV-CPC-N and HGF that was found in EV-CPC-P, CPC-P treated with EV-CM had an increased proliferation rate, as demonstrated by the ratio of cells in active phases of cell cycle (Figure 5). Furthermore, the cargo delivered by EV-CPC-N exerted a cardioprotective effect on CPC-P as supported by the evidence of an increased resistance to apoptosis induced by oxidative stress (Figure 5). Therefore, EV-CPC-N are preferable candidates for treating cardiovascular diseases by an intriguingly appealing cell-free approach, as they show a best potential of delivering signals to prompt differentiation to cardiomyocyte and of exerting a positive effect on CPC proliferation and protection.

It is well-researched that the fate of the cell is determined by coordinated and dynamic interactions among several factors, acting in a defined microenvironment (Romano et al., 2021; Cotrufo et al., 2005). ECM accounts for approximately 24% of myocardial volume and network rearrangement and enlargement is an essential component of cardiac remodeling at various pathological stages (Kapelko, 2001). The ECM elasticity determines stem cell lineage specification, expansion, and differentiation (Engler et al., 2006; Mishra et al., 2012) and, in the heart, ECM provides a framework that exhibits the mechanical properties required for the differentiation of cardiac stem cells (Eitan et al., 2010). Stem cells, indeed, are highly sensitive to extracellular signals that play a critical role in maintenance of their characteristics and interplay with somatic cells (Quesenberry et al., 2015; Belviso et al., 2020c). Cell-cell and cell-matrix cross talk plays a central role both in cardiac homeostasis and in adaptive responses of the heart to stress and extracellular matrix, and ECM proteins can drive cardiac tissue regeneration (Castaldo et al., 2008; Boffito et al., 2018) described in adult human heart in infarction or pressure overload (Cesselli et al., 2011). Chronic adaptive responses to stress, referred to as cardiac remodeling, include interstitial fibrosis, cardiomyocytes hypertrophy, and changes in contractility and blood vessel density (Qing-Qing et al., 2017). Post MI, the proliferation of fibroblasts leads to the formation of collagen-rich, non-contractile, scar tissue (Hill and Olson, 2008) that, when combined with the extensive cardiomyocyte death (Burchfield et al., 2013), leads to long-term systolic dysfunction. In the damaged heart, fibroblasts are stimulated by cytokines such as TGF-β, which leads to exacerbation of extracellular matrix production (Brigstock, 2010) and enhanced fibrosis (Liu et al., 2011). CPC have been previously shown to exert potentially anti-fibrotic effects by transferring EVs to fibroblasts and by promoting cardiac myocyte survival *in vitro* (Wang et al., 2015). Consistently, when delivered to CF-P in culture through EV-CM, EV-CPC-N affected the synthesis and deposition of ECM proteins (Figure 7). Specifically, while collagen type I and III, laminin and tenascin synthesis remained unaltered when compared to the control group, the synthesis of collagen type IV and fibronectin significantly increased in CF-P treated with EV-CPC-N. Remarkably, fibronectin is known to drive collagen assembly (Sevilla et al., 2013) and to promote CPC proliferation and protection, as demonstrated by an impaired CPC expansion and reduced healing process after myocardial infarction in fibronectin knockout mice (Singh et al., 2010). Additionally, we observed *in vitro* the fibronectin disarrangement that has been previously described in post-ischemic cardiac fibrosis (Berezin and Berezin, 2020) and, interestingly, the administration of EV-CPC-N to CF-P *in vitro* conferred to fibronectin deposition an organized fibrillary pattern (Figure 8) inducing to speculate that EV-CPC-N positively affect the extracellular compartment during the response to injury.

## Conclusion

Our findings are the first to our knowledge that provide evidence in support of combined beneficial effects of EVs released by healthy human CPC on both resident supporting cells and CPC of post-ischemic adult human heart (Zhang et al., 2016). Notably, the positive outcomes emerged for EV-CPC-N are encouraging, highlighting they could be also appropriate and healing for clinical use instead of CPCs themselves.

### Limitation of the Study

The findings of this study have to be seen in light of a relevant limitation.

Indeed, *in vivo* studies, aimed at investigating the feasibility of cardiac regenerative therapies based on the use of EV-CPC and to explore the potential of EV-CPC as cost-effective off-the-shelf alternative therapeutic modality, are needed. Although based on *in vitro* experiments, evidence emerging from our study offers an intriguing perspective on the underlying mechanisms responsible for effects of CPC-based therapy of myocardial infarction (Ostovaneh et al., 2021).

## Data Availability

All data supporting the findings of this study are available within the article and its Supplementary Information; source data for the figures in this study are available from the authors upon request.

## Abbreviations

CFs, Cardiac Fibroblasts; CF-P, Cardiac Fibroblasts from Pathological Hearts; CPC, Cardiac Progenitor Cells; CPC-N, Cardiac Progenitor Cells from Normal Hearts; CPC-P, Cardiac Progenitor Cells from Pathological Hearts; EV-CM, Conditioned Medium; EVs, Extracellular Vesicles; EV-CPC-N, Extracellular Vesicles from Cardiac Progenitor Cells of Normal Hearts; EV-CPC-P, Extracellular Vesicles from Cardiac Progenitor Cells of Pathological Hearts; ECM, Extracellular Matrix; GFs, Growth Factors.
