# Supplementary material for: Human Cardiac Progenitor Cell-Derived Extracellular Vesicles Exhibit Promising Potential for Supporting Cardiac Repair in Vitro
Source: Front Physiol. 2022 May 20;13:879046. doi: 10.3389/fphys.2022.879046 (PMC9163838; doi:10.3389/fphys.2022.879046)
Supplement: Supplementary file 1 [file DataSheet1.PDF]

Supplementary Material

Supplementary Tables

Table 1: Patients' main clinical information (T2DM: Type 2 diabetes mellitus; AH: Arterial hypertension; PAH: Pulmonary arterial hypertension; CKD: Chronic kidney disease; COPD: Chronic obstructive pulmonary disease; BPH: benign prostatic hyperplasia; GIST: Gastrointestinal stromal tumor).

Patient	Age	Duration of disease (days)	T2DM	AH	PAH	CKD	COPD	Other	Therapy
**01.**	59	250	Y	Y	N	N	N	-	Furosemide, bisoprolol, amlodipine, allopurinol, acetylsalicylic acid, pantoprazole, insulin, gabapentin, quetiapine
**02.**	55	359	N	Y	N	N	N	-	Furosemide, omeprazole, ursodeoxycholic acid, carvedilol, digoxin, canrenoate, warfarin
**03.**	65	709	N	Y	N	N	N	-	Furosemide, bisoprolol, allopurinol, ramipril, canrenoate, atorvastatin, amiodarone, levotiroxine
**04.**	59	21	N	Y	Y	N	N	-	Furosemide, carvedilol, spirolactone, digoxin, atorvastatin, amiodarone, warfarin
**05.**	56	67	N	Y	N	N	N	-	Furosemide, carvedilol, allopurinol, warfarin, canrenoate, alprazolam
**06.**	58	1401	Y	Y	N	Y	N	-	Furosemide, bisoprolol, canrenoate, telmisartan, atorvastatin, allopurinol, acarbose, warfarin
**07.**	60	681	Y	N	N	Y	N	-	Furosemide, canrenoate, acetylsalicylic acid,

									omeprazole, metolazone, allopurinol, warfarin
**08.**	42	386	N	Y	Y	N	N	-	Furosemide, canrenoate, bisoprolol, warfarin
**09.**	56	196	N	N	N	N	N	-	Furosemide, pantoprazole, canrenoate, allopurinol, carvedilol
**10.**	62	32	Y	Y	Y	N	N	-	Furosemide, allopurinol, bisoprolol, amiodarone, insulin, captopril, warfarin
**11.**	60	721	Y	N	N	N	Y	-	Allopurinol, insulin, acetylsalicylic acid, ivabradine
**12.**	54	87	N	Y	N	N	N	BPH	Furosemide, pantoprazole, bisoprolol, canrenoate, acetylsalicylic acid, sacubitril/valsartan, amiodarone, ranolazine
**13.**	51	179	N	N	Y	N	N	-	Furosemide, spirolactone, omeprazole, ramipril, carvedilol, allopurinol
**14.**	61	425	N	Y	Y	N	N	Amiodarone-induced dysthyroidism	Torasemide, atorvastatin, acetylsalicylic acid, digoxin, spirolactone, sildenafil, warfarin
**15.**	58	80	N	N	Y	N	Y	GIST, BPH	Furosemide, carvedilol, canrenoate, amiodarone, ranitidine, digoxin, ivabradine, serenoa repens, sulbactam/ampicillin, warfarin
**16.**	46	1721	N	N	Y	N	N	-	Furosemide, pantoprazole, atorvastatin, bisoprolol, allopurinol, ranolazine, sacubitril/valsartan

**17.**	57	212	N	N	Y	N	N	-	Furosemide, spirolactone, amiodarone, folic acid, bisoprolol
**18.**	53	814	Y	N	Y	Y	N	-	Furosemide, carvedilol, canrenoate, atorvastatin, enalapril, insulin, ezetimibe, acetylsalicylic acid, ivabradine
**19.**	50	2148	N	Y	N	N	N	-	Furosemide, bisoprolol, enalapril, ivabradine, digoxin, atorvastatin, allopurinol, pantoprazole, amiodarone, warfarin
**20.**	58	33	N	Y	N	N	N	-	Furosemide, bisoprolol, acetylsalicylic acid, allopurinol

Table 2: List and results of growth factor content analyses performed by protein array on EVs from CPC-N and CPC-P.

Growth Factor	CPC-N (mean ± SEM)	CPC-P (mean ± SEM)	P value
AREG	0.0067 ± 0.0033	0.0125 ± 0.0070	0.4586
b FGF	0.0800 ± 0.0155	0.0708 ± 0.0250	0.7589
b NGF	0.0333 ± 0.0114	0.0483 ± 0.0170	0.4711
EGF	0.2800 ± 0.0410	0.1589 ± 0.0259	0.0264
EGFR	0.0083 ± 0.0034	0.0025 ± 0.0018	0.1474
FGF-4	0.2058 ± 0.0823	0.2308 ± 0.0623	0.8119
FGF-6	0.4992 ± 0.1169	0.1183 ± 0.0637	0.0091
FGF-7	0.6983 ± 0.1006	0.2092 ± 0.0826	0.0011
G-CSF	0.0225 ± 0.0156	0.0750 ± 0.0291	0.1266
GDNF	0.1083 ± 0.0491	0.1967 ± 0.0449	0.1979

GM CSF	0.0250 ± 0.0172	0.0300 ± 0.0104	0.8062
HB EGF	0.0708 ± 0.0214	0.2433 ± 0.0419	0.0014
HGF	0.0050 ± 0.0034	0.1200 ± 0.0442	0.0165
IGFBP1	0.1592 ± 0.0326	0.2233 ± 0.0554	0.3287
IGFBP2	0.1083 ± 0.0231	0.2917 ± 0.0552	0.0057
IGFBP3	0.0533 ± 0.0215	0.2425 ± 0.0643	0.0533
IGFBP4	0.0183 ± 0.0074	0.0650 ± 0.0259	0.0967
IGFBP6	0.0233 ± 0.0115	0.0417 ± 0.0189	0.4156
IGF1	0.4633 ± 0.0303	0.3000 ± 0.0345	0.0026
IGF-1sR	1.183 ± 0.1215	0.5625 ± 0.0767	0.0003
IGF-2	0.2158 ± 0.0494	0.6717 ± 0.0446	<0.0001
M-CSF	0.0600 ± 0.0293	0.4258 ± 0.0514	<0.0001
M-CSFR	0.0750 ± 0.0272	0.3158 ± 0.0501	0.0004
NT3	0.0750 ± 0.0219	0.3458 ± 0.0517	<0.0001
NT4	0.0133 ± 0.0056	0.2117 ± 0.0496	0.0006
PDGFR alpha	0.0233 ± 0.0181	0.2450 ± 0.0539	0.0008
PDGFRbeta	0.0608 ± 0.0429	0.3450 ± 0.0552	0.0005
PDGF AA	0.5078 ± 0.0791	0.9372 ± 0.0373	<0.0001
PDGFAB	0.1992 ± 0.676	0.5067 ± 0.0660	0.0036
PDGF BB	0.1908 ± 0.0865	0.3725 ± 0.0694	0.1157
PLGF	0.3092 ± 0.1263	0.5150 ± 0.0679	0.1653
SCF	1.057 ± 0.0816	0.8467 ± 0.0242	0.0218
SCFR	0.0233 ± 0.0159	0.2883 ± 0.0512	<0.0001
TGF alpha	0.0042 ± 0.0042	0.1875 ± 0.0489	0.0011
TGF beta	0.0133 ± 0.0093	0.1825 ± 0.0387	0.0003
TGFbeta 2	0.0150 ± 0.0110	0.2342 ± 0.0424	<0.0001
TGFbeta3	0.0033 ± 0.0026	0.3408 ± 0.0501	<0.0001

VEGF	0.0058 ± 0.0050	0.2667 ± 0.0504	<0.0001
VEGFR2	0.0325 ± 0.0255	0.3567 ± 0.0662	0.0002
VEGFR3	0.1858 ± 0.0424	0.6758 ± 0.0765	<0.0001
VEGFD	0.0708 ± 0.0474	0.5092 ± 0.0527	<0.0001
